# Bioptic Study of Left and Right Atrial Interstitium in Cardiac Patients with and without Atrial Fibrillation: Interatrial but Not Rhythm-Based Differences

**DOI:** 10.1371/journal.pone.0129124

**Published:** 2015-06-12

**Authors:** Natalia Smorodinova, Lucie Lantová, Martin Bláha, Vojtěch Melenovský, Jan Hanzelka, Jan Pirk, Josef Kautzner, Tomáš Kučera

**Affiliations:** 1 Institute of Histology and Embryology, The First Faculty of Medicine, Charles University in Prague, Prague, Czech Republic; 2 Institute for Clinical and Experimental Medicine-IKEM, Department of Cardiology, Prague, Czech Republic; 3 Institute for Clinical and Experimental Medicine-IKEM, Department of Cardiovascular Surgery, Prague, Czech Republic; Gent University, BELGIUM

## Abstract

One of the generally recognized factors contributing to the initiation and maintenance of atrial fibrillation (AF) is structural remodeling of the myocardium that affects both atrial cardiomyocytes as well as interstitium. The goal of this study was to characterize morphologically and functionally interstitium of atria in patients with AF or in sinus rhythm (SR) who were indicated to heart surgery. Patient population consisted of 46 subjects (19 with long-term persistent AF, and 27 in SR) undergoing coronary bypass or valve surgery. Peroperative bioptic samples of the left and the right atria were examined using immunohistochemistry to visualize and quantify collagen I, collagen III, elastin, desmin, smooth muscle actin, endothelium and Vascular Endothelial Growth Factor (VEGF). The content of interstitial elastin, collagen I, and collagen III in atrial tissue was similar in AF and SR groups. However, the right atrium was more than twofold more abundant in elastin as compared with the left atrium and similar difference was found for collagen I and III. The right atrium showed also higher VEGF expression and lower microvascular density as compared to the left atrium. No significant changes in atrial extracellular matrix fiber content, microvascular density and angiogenic signaling, attributable to AF, were found in this cohort of patients with structural heart disease. This finding suggests that interstitial fibrosis and other morphological changes in atrial tissue are rather linked to structural heart disease than to AF per se. Significant regional differences in interstitial structure between right and left atrium is a novel observation that deserves further investigation.

## Introduction

Atrial fibrillation (AF) is one of the most common cardiac arrhythmias in human [1]. Rarely, AF may occur in patients without traditional structural heart disease and without conventional risk factors (so-called „lone AF“) [2]. Experimental models showed that AF itself results in variable degree of structural remodeling of atrial tissue that beget further maintenance of arrhythmia and may explain its progressive character [3, 4]. However, clinical evidence to support this view is scarce and only few studies have identified significant structural changes in patients with lone AF [5, 6]. On the other hand, AF is more frequently associated with atrial hypertension and/or with different types of structural heart disease that lead to progressive atrial enlargement and stretch [3, 7]. Altered hemodynamics and/or disease itself result also in variable degree of structural changes in atrial tissue that can contribute both to triggering and maintenance of AF.

It is generally accepted that a characteristic hallmark of structural remodeling and an important morphological substrate of AF is interstitial fibrosis. Morphologically, fibrosis is defined as an increased fraction of extracellular matrix, in particular collagen fibers [8]. In addition, collagen, elastin and reticular fibers are important components of the atrial wall that determine its mechanical properties such as compliance [9]. Less is known about possible regional differences of these structures. Similarly, changes in microvasculature due to tissue hypoxia and their relationship to AF have been studied only sporadically [10, 11].

The objective of the present study was to perform a comprehensive analysis of atrial morphology with the aim to compare tissue samples from the left and right atrium harvested during open-heart surgery in patients with sinus rhythm (SR) or AF.

## Materials and Methods

### Patients

The study group consisted of 46 patients (19 with long-term persistent AF and 27 in SR) who underwent cardiac surgery at the Institute for Clinical and Experimental Medicine in Prague. In both AF and SR groups, there were patients who underwent coronary artery bypass surgery or valve surgery (S1 Table). The study protocol was approved by the ethics committee of the Institute for Clinical and Experimental Medicine in Prague, and the study conformed to the principles outlined in the Declaration of Helsinki. All patients included in the study signed a written informed consent.

### Tissue sampling

Tissue samples were obtained during open-heart surgery. A piece of atrial tissue of a maximum size of 5*5 mm was excised as a transverse section from the right appendage, the left free atrial wall and from the left appendage. Samples were immediately fixed with 4% formaldehyde. No complication occurred as a consequence of tissue sampling.

### Histological, immunohistochemical and immunofluorescence staining

Harvested atrial samples were embedded into paraffin and cut to 7μm thick tissue sections. Sections were subsequently dewaxed and rehydrated. Deparaffinized tissue sections were histologically examined using routine hematoxylin-eosin staining. Immunohistochemistry was used to visualize collagen I, collagen III, elastin, desmin, smooth muscle actin and VEGF in these samples (S2 Table). Three-step immunoperoxidase detection was also performed on the paraffin sections. After antigen retrieval with citric buffer (pH = 6.0) or TRIS+EDTA buffer (pH = 9.0), the endogenous peroxidase activity and the non-specific antibody binding sites were blocked with 5% swine or goat serum in PBS. Next, the sections were incubated with a primary antibody overnight at 4° or for 60 min at room temperature. Visualization of antibody binding was performed using a LSAB+ peroxidase kit (Dako, Glostrup, Denmark).

To detect capillaries within the atrial tissue, we used biotinylated Ulex europaeus agglutinin I (Vector Laboratories, USA) that was applied (1:200) overnight at 4°C. Visualization of lectin binding was performed using the Vectastain Elite ABC kit standard (Vector Laboratories, USA).

For assessment of desmin expression in pericytes and other cells, we used immunofluorescence staining with the use of primary monoclonal mouse anti-human desmin 1:100 in 1% goat serum in PBS and secondary anti-mouse Alexa Fluor 532 (Invitrogen, Life Technologies) 1:500 in 5% goat serum in PBS antibodies.

To detect pericytes for microvessel pericyte coverage index assessment, we used monoclonal mouse anti-human smooth muscle actin antibody and biotinylated Ulex europaeus agglutinin I (Vector Laboratories, USA) diluted 1:200 in 1% goat serum in PBS. Streptavidin Alexa Fluor 488 (Invitrogen, Life Technologies) 1:100 and anti-mouse Alexa Fluor 532 (Invitrogen, Life Technologies) 1:500 in 5% goat serum in PBS were used in the next step. The nuclei were stained with DAPI 1:1000. Negative controls were used for all experiments.

### Histomorphometry

To quantify collagen I, collagen III and elastin volume fraction, we used the program Image J 1.44p (National Institutes of Health, USA). Images for quantification were collected by systematic uniform random sampling of tissue sections using the 40x dry objective of a Leica DMLB microscope (Leica Microsystems GmbH, Wetzlar, Germany). From each sample of atrial myocardium, 10 images from a single section were recorded. Individual recordings were separated from each other by 1 image frame. Images were converted to an 8-bit grey scale format and the threshold was set above the background staining intensity. Immunolabelled areas were automatically detected and area fraction was calculated for collagen I, collagen III and elastin. Only endomysial collagen and elastin fibers were quantified in this analysis. Perimysial connective tissue separating sheets of myocardial cells was excluded from quantitation as well as epicardial and endocardial connective tissue.

To quantify microvascular density Ulex europaeus agglutinin I staining was used. Measurements were made using Image J 1.44p (National Institutes of Health, USA). Images for quantification were collected as described above and microvascular density was calculated as the number of capillaries per area unit.

VEGF expression was analyzed semiquantitatively. Microphotography and image sampling was performed as described above. We evaluated the intensity of positive cardiomyocytes, adipocytes, mesothelial cells and capillaries. The intensity score consisted of four grades (0–3), with 0 representing no staining and 3 denoting the maximum staining effect.

To quantify microvessel pericyte coverage index, we used immunofluorescence staining and analyzed microvessel pericyte coverage of capillaries in the atrial myocardium. Pericytes were identified as cells immunoreactive for smooth muscle actin and characterized as elongated cells surrounding capillaries. Capillaries were positive for Ulex europaeus agglutinin I and marked in green. Random areas were photographed using a Confocal Laser Scanning Biological Microscope Fluoview FV1000 Spectral Type (Olympus) equipped with a 40× oil immersion objective and analyzed by Olympus Fluoview 10-ASW 3.1 software. The percentage of capillaries with pericyte coverage was counted.

Those who performed image analysis and scoring of immunohistochemical labeling were blinded to patient characteristics.

### Statistical Analysis

The values are expressed as a mean±SD. Comparison between both groups was performed using a non-parametric test—Mann–Whitney U test. A value of *P* < 0.05 was considered significant.

## Results

### Patient characteristics

Clinical characteristics of the patient population are listed in S1 Table. Patients from AF group were on average 7 years older, had more pronounced mitral regurgitation and higher left and right atrial volumes. On the other hand, SR patients had more often coronary artery disease. Left atrial volume was on average 121.3±62.5 ml in patients with AF, while the average left atrial volume was 73.0±28.5 ml in SR group. A smaller difference was found in the right atrial volumes (84.2±39.5 ml in AF group vs. 61.3±23.8 ml in SR group). In contrast to these atrial parameters, LV diameter did not differ significantly between patients with AF and patients with SR (55.4±8.0 mm in AF group vs. 53.0±6.1 mm in SR group). There was no difference in NYHA class between both patient groups (2.31±0.54 in AF group vs. 2.33±0.64 in SR group).

### Collagen I and collagen III in atrial myocardium

**Fig 1:**
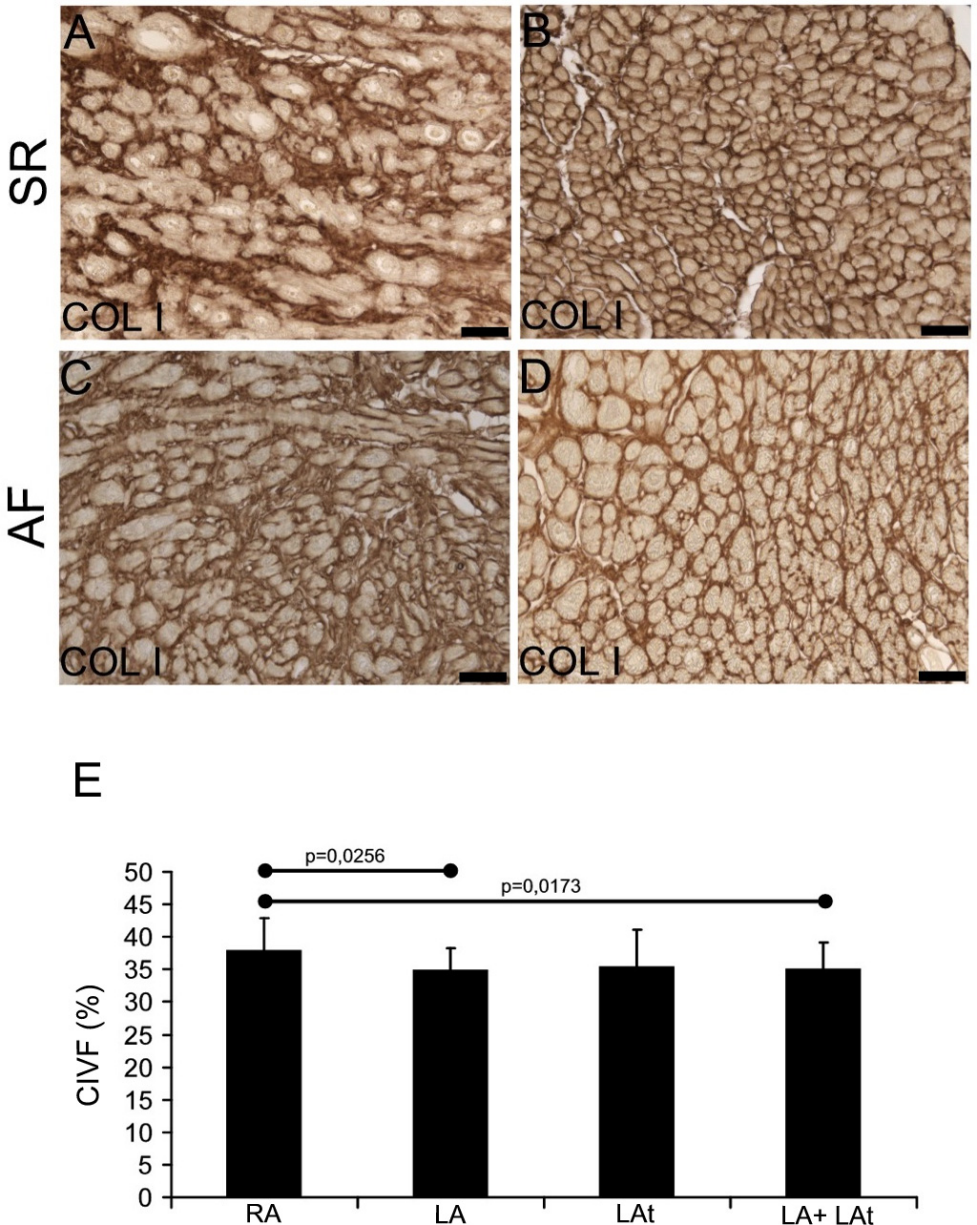
Collagen I in the atrial myocardium. A-D: Immunohistochemical reaction shows collagen I in endomysial extracellular matrix. In atria of both patient groups (SR in A, B and AF in C, D) it is possible to detect higher (A, C) as well as lower (B, D) amount of collagen I-positive ECM. Immunoperoxidase reaction with DAB as a substrate (brown precipitate). No nuclear counterstaining. For all images scale bar = 50μm. (E) A graph showing the result of quantification of collagen I volume fraction (CIVF) in atrial myocardial samples. A comparison between different anatomical locations is shown: right appendage–RA (n = 37), left appendage–LA (n = 18), left atrium—LAt (n = 8), LA+LAt (n = 26).

**Fig 2:**
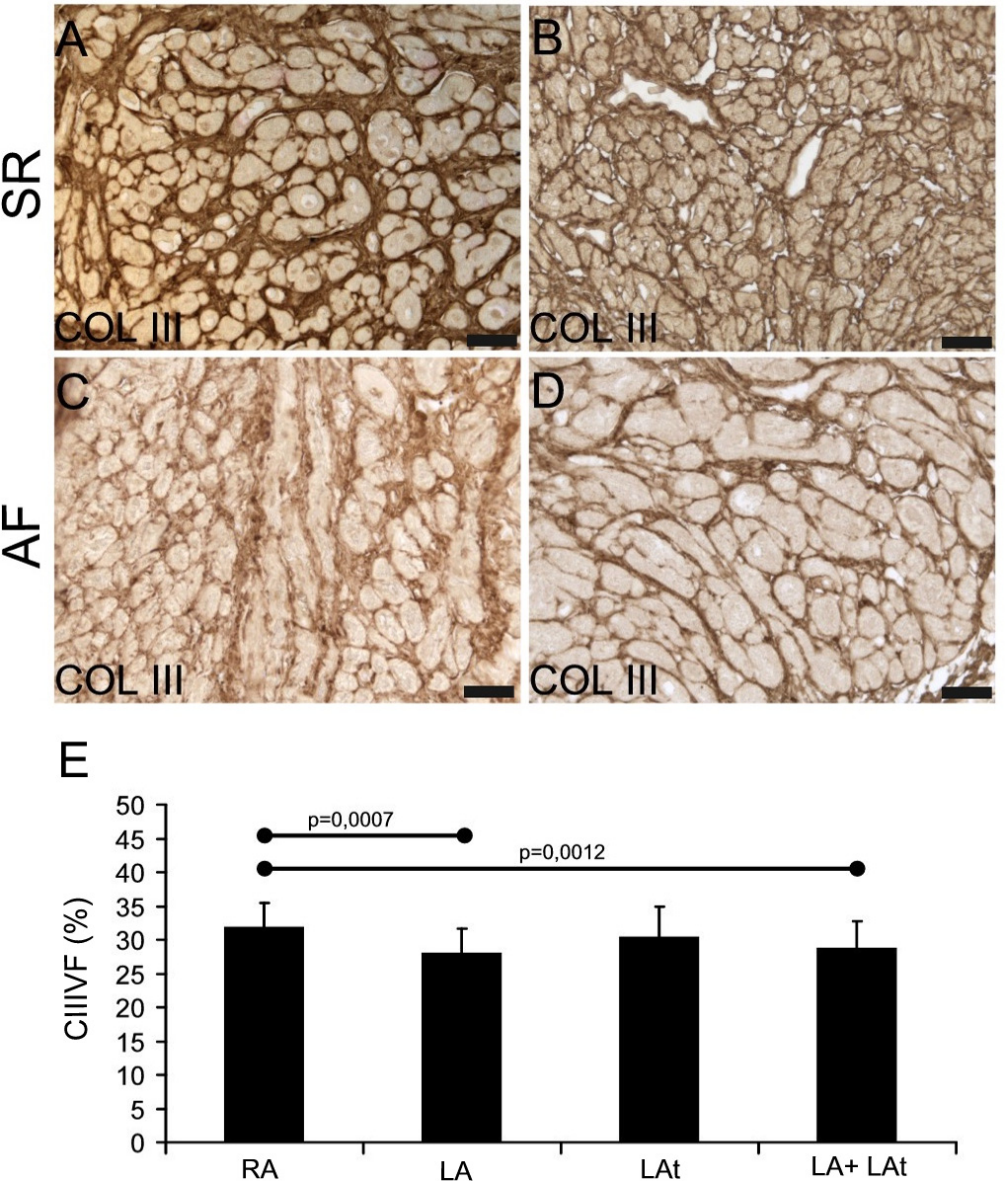
Collagen III in the atrial myocardium. A-D: Immunohistochemical reaction shows collagen III in endomysial and partly also perimysial extracellular matrix. In atria of both patient groups (SR in A, B and AF in C, D) it is possible to detect higher (A, C) as well as lower (B, D) amount of collagen III-positive ECM. Immunoperoxidase reaction with DAB as a substrate (brown precipitate). No nuclear counterstaining. For all images scale bar = 50μm. (E) A graph showing the result of quantification of collagen III volume fraction (CIIIVF) in atrial myocardial samples. A comparison between different anatomical locations is shown: right appendage–RA (n = 37), left appendage–LA (n = 19), left atrium—LAt (n = 9), LA+LAt (n = 28).

To compare the level of fibrosis in atrial tissue samples from patients with SR and AF, we focused first on collagen I in the atrial endomysium, using immunohistochemical detection. Variable amount of collagen I was found in samples from both patient groups (Fig 1A–1D). In samples with low amount of collagen I, the immunoreactivity was found evenly distributed in thin endomysial layer, surrounding individual cardiomyocytes (Fig 1B–1D). In other samples, thicker layers of collagen I were identified, surrounding individual cardiomyocytes or groups of cardiomyocytes isolated by a thick layer of collagen-I positive ECM (Fig 1A–1C). Volume fraction of collagen I was similar when comparing samples from AF and SR group (S3 Table). Next we compared collagen I volume fraction in samples from different anatomical locations and found that there was significantly higher collagen I volume fraction in the right appendage compared to the left appendage as well as compared to pooled samples from the left atrial free wall and the left appendage (Fig 1E). Distribution and pattern of collagen III immunoreactivity were similar to that of collagen I (Fig 2A–2D). Volume fraction of collagen III was similar when comparing samples from AF and SR group (S3 Table). There was significantly higher collagen III volume fraction in the right appendage compared to the left one as well as compared to pooled samples from the left atrial free wall and the left appendage (Fig 2E).

### Elastin in atrial myocardium

**Fig 3:**
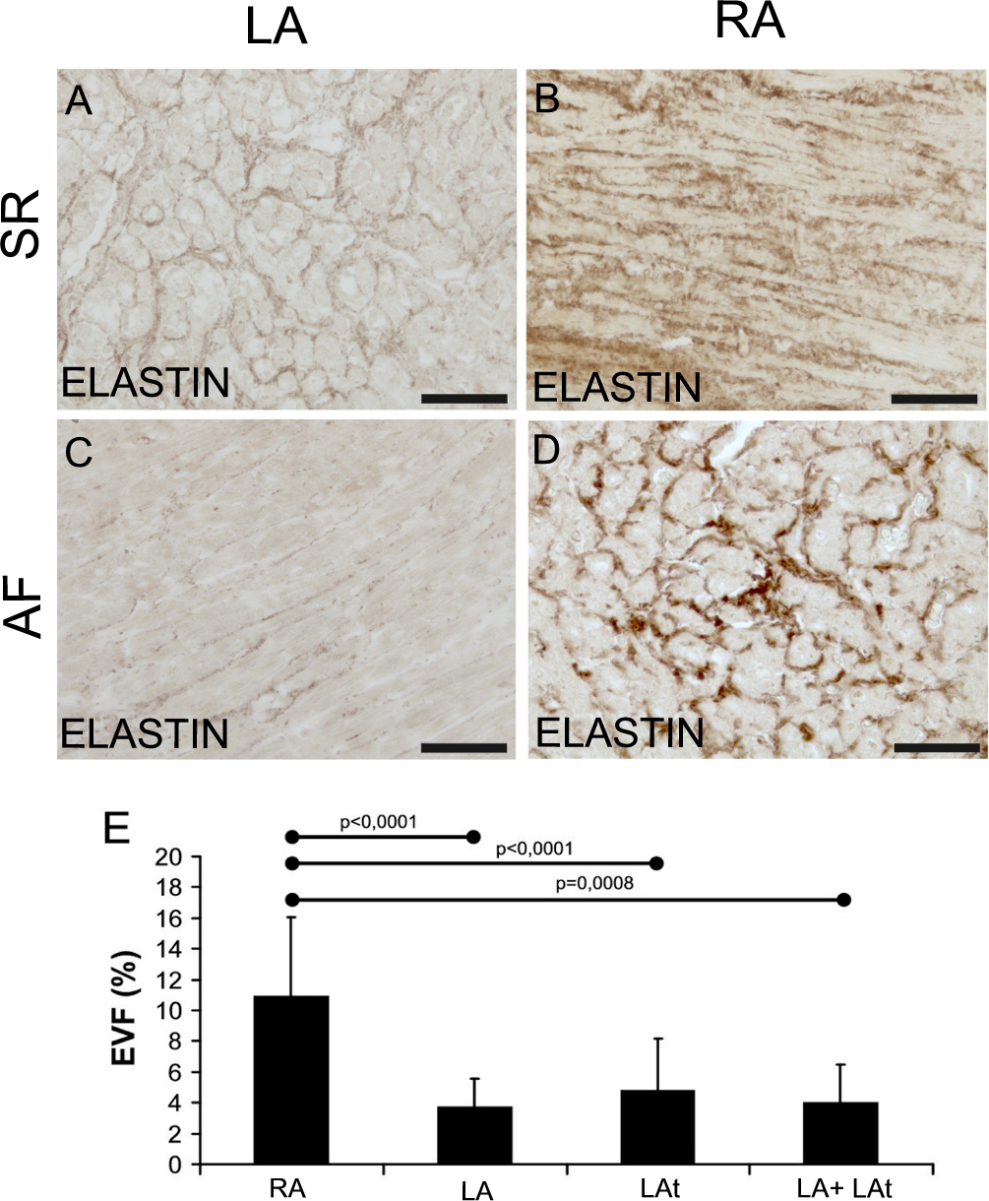
Elastin in the atrial myocardium. Immunohistochemical detection of elastin in samples from patients with sinus rhythm (A, B) and in samples from patients with atrial fibrillation (C, D). EVF (elastin volume fraction) is the percentage of elastin fibers in morphometrically evaluated myocardial area. Representative images of left appendage myocardium with low EVF (A, C) and images from right appendage show high EVF (B, D) Immunoperoxidase reaction with DAB as a substrate (brown precipitate). No nuclear counterstaining. Scale bar in A-D = 100μm. E: A graph showing atrial EVF of all patients with sinus rhythm and atrial fibrillation dependent on localization. RA–right appendage (n = 26), LA–left appendage (n = 12), LAt–left atrium (n = 4), LA + LAt (n = 16).

Elastin was detected in human atrial samples in variable amount (Fig 3). Some samples contained low amount of elastin (Fig 3A–3C), while other were rich in elastin (Fig 3B–3D). Quantitative morphometric analysis of elastin volume fraction revealed no significant difference between samples from patients with AF and samples from SR patients (S3 Table). However, elastin volume fraction was generally lower than collagen I volume fraction and collagen III volume fraction. Compared to collagen content (collagen I and III volume fraction), similar but more pronounced differences in elastin volume fraction were discovered among three anatomical locations (compare Fig 1E and Fig 2E with Fig 3E). Significantly higher content of elastin was found in the right appendage compared to the left one, the left atrial free wall or pooled samples from the left atrial free wall and the left appendage (Fig 3E).

### VEGF in atrial myocardium

**Fig 4:**
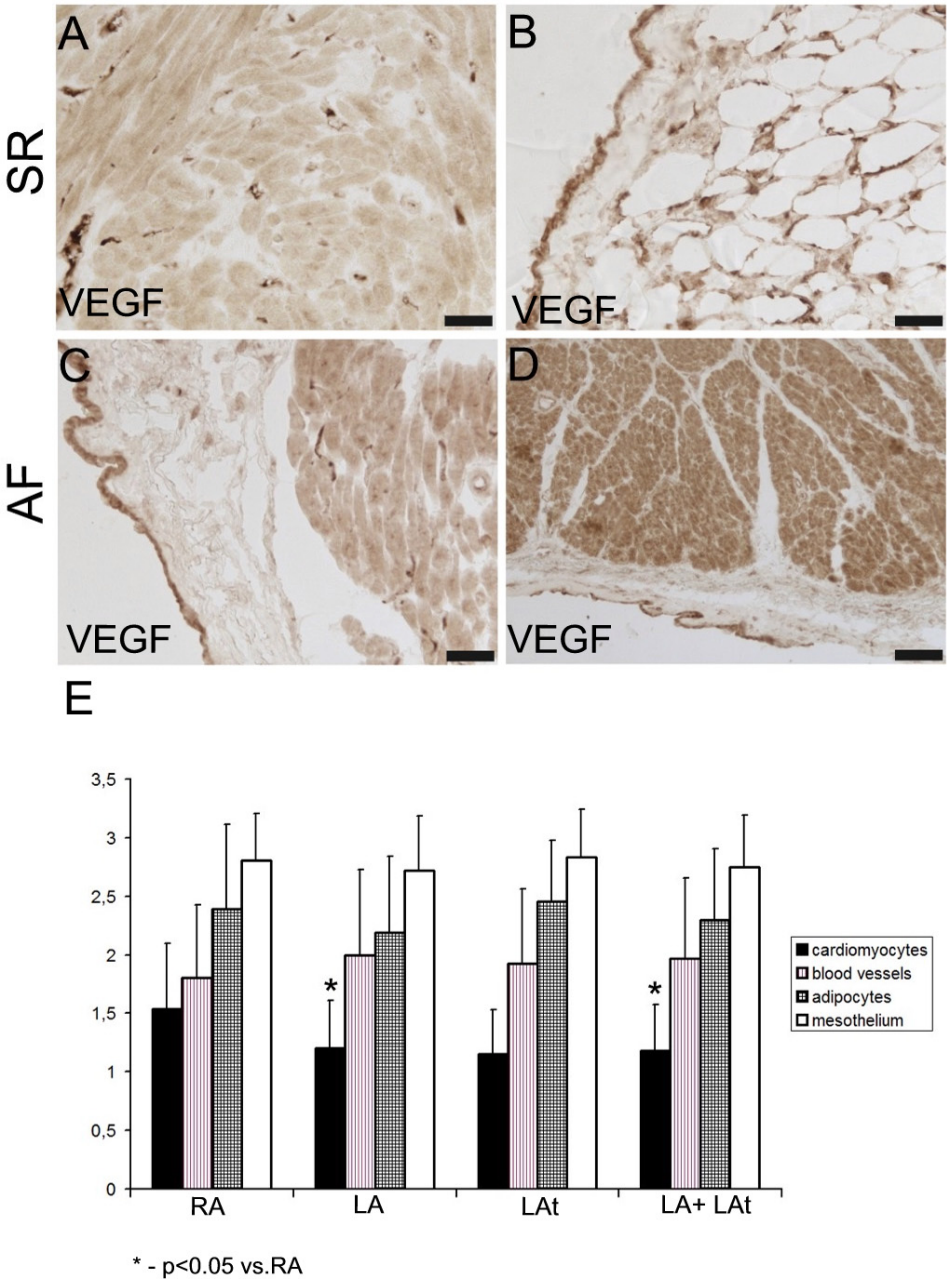
Expression of VEGF in atrial myocardium from patients with sinus rhythm (SR) and atrial fibrillation (AF). A, B: Atrial samples from patients with SR. (A) A strong immunoreactivity for VEGF is localized to the capillaries, while cardiomyocytes display rather low level of VEGF expression. (B) High level of VEGF immunoreactivity in mesothelial cells and in adipocytes of epicardium. C, D: Atrial samples from patients with AF. (C) A strong immunoreactivity for VEGF is localized to mesothelium. There are VEGF-positive capillaries and moderately positive cardiomyocytes in the atrial myocardium. (D) A strong VEGF immunoreactivity in the myocardium (mainly cardiomyocytes) and in mesothelial cells. Scale bar in A-D = 50μm. (E) A graph showing a result of semiquantitative analysis of VEGF immunoreactivity in the atrial samples from all patients as described in Methods (score 0–3). A comparison between different structures from different anatomical locations is shown: RA–right appendage, LA–left appendage, LAt–left atrium.

VEGF immunoreactivity was discovered in all atrial tissue samples that were examined. We focused not only on the myocardium but also on endocardial and epicardial layer. In the epicardium, VEGF immunoreactivity was found in mesothelial cells and adipocytes as well as in cardiomyocytes and blood vessels, mainly capillaries (Fig 4A–4D). The expression of VEGF was variable. Semiquantitative analysis of VEGF expression revealed the strongest expression in the epicardial mesothelium. The level of VEGF immunoreactivity was similar among patients with AF and SR (S4 Table). A comparison of VEGF expression by cardiomyocytes based on the anatomical location of samples from all patients revealed higher VEGF expression in the right appendage compared to the left one (Fig 4E). The difference against the right atrial appendage was also found for pooled samples from the left appendage and left atrial free wall (Fig 4E).

### Microvascular density in atrial myocardium

**Fig 5:**
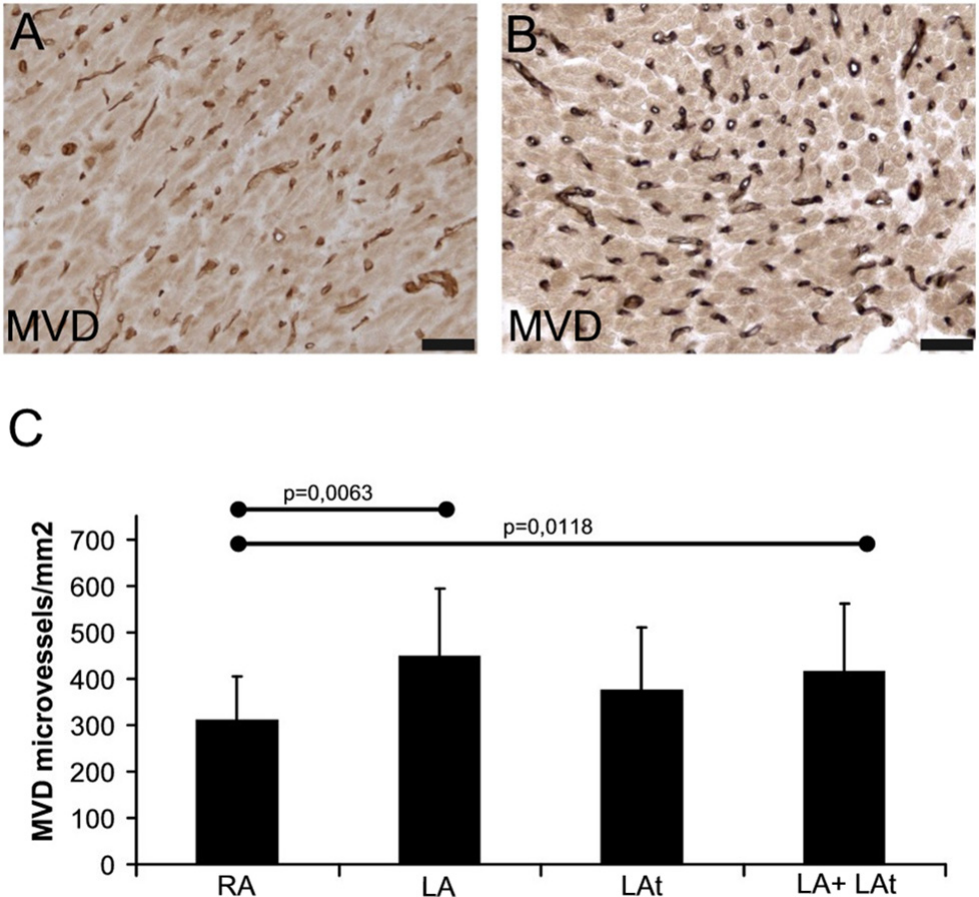
Quantification of microvascular density. Histochemical visualization of microvasculature using UEA-lectin binding in atrial myocardium from patients with sinus rhythm (SR) (A) and atrial fibrillation (AF) (B). Peroxidase reaction with DAB as a substrate (brown precipitate). No nuclear counterstaining. Scale bar in A-B = 50μm. (C) A graph showing results of quantification of microvascular density (MVD) in atrial samples of all patients as described in Methods. RA–right appendage (n = 24), LA–left appendage (n = 12), LAt–left atrium (n = 10), LA + LAt (n = 22).

Microvasculature was analyzed in tissue samples from the same patient groups as VEGF expression. Using UEA-lectin blood vessel endothelium was visualized in atrial myocardium (Fig 5A and 5B); microvascular density was quantified in samples of atria from both AF and SR groups. No statistical difference was found in the level of microvascular density between patients with AF and subjects in SR (S3 Table). Microvascular density in the right appendage was significantly lower compared both to the left appendage alone as well as to the left appendage and left atrial free wall (Fig 5C).

### Atrial microvessel pericyte coverage

**Fig 6:**
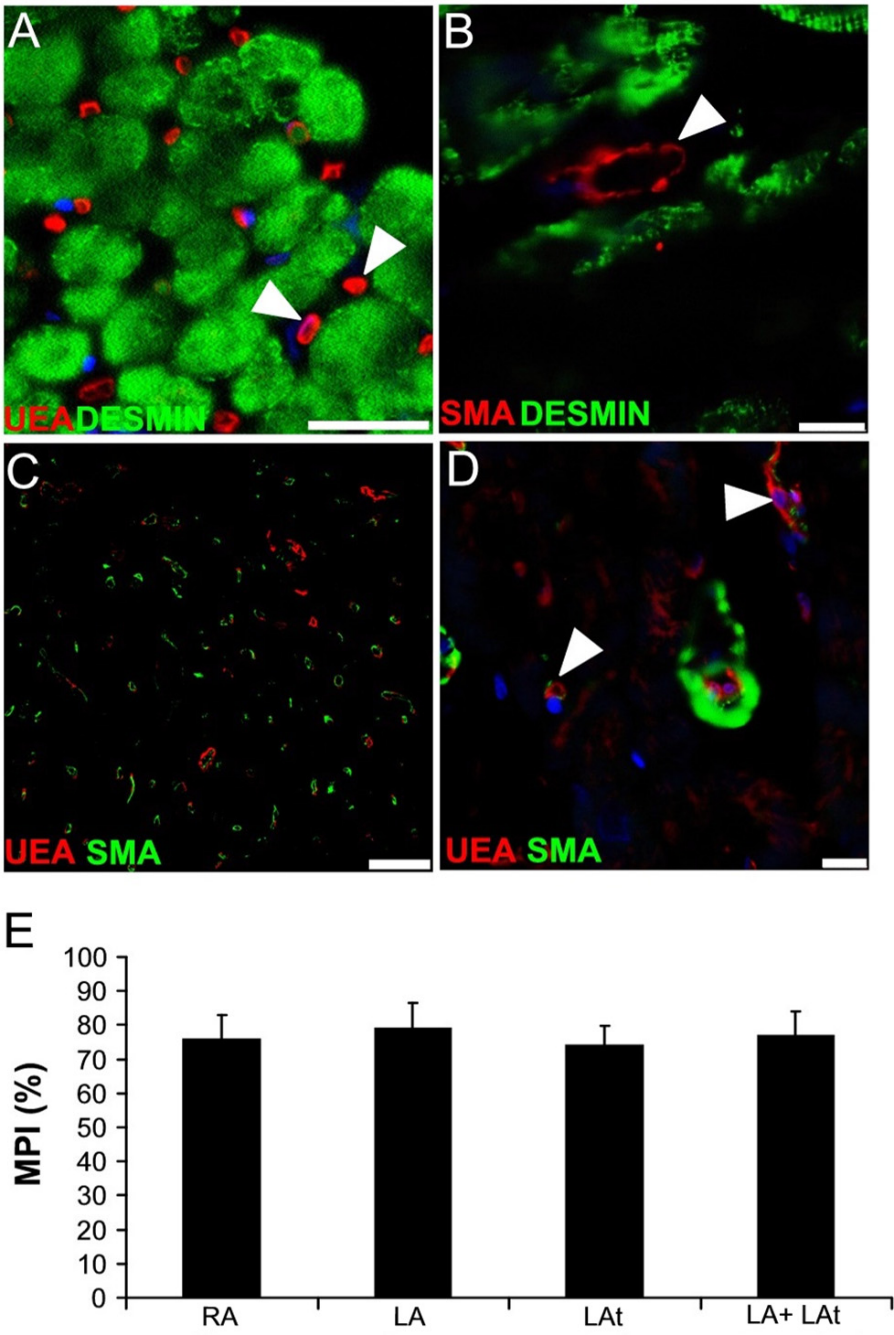
Microvessel pericyte coverage in atria of patients with sinus rhythm (SR) and atrial fibrillation (AF). (A-D) Confocal images of human atria. (A) Immunodetection of desmin (green) shows its expression in cardiomyocytes. Capillaries labeled with UEA-lectin (red) are devoid of desmin immunoreactivity (arrowheads). Scale bar = 50μm. (B) Desmin is present in cardiomyocytes, while SMA-positive capillary pericyte (arrowhead) does not display desmin signal. Scale bar = 10μm. (C) A representative section of myocardium used for the quantitative analysis of microvessel pericyte coverage index. Blood vessel endothelium is labeled using UEA lectin (red) and pericytes are visualized using anti-SMA antibody (green). Scale bar = 100μm. (D) A high magnification image shows SMA-positive smooth muscle cells surrounding a small arteriole and also SMA-positive pericytes in association with the capillaries (arrowheads). Scale bar = 25μm. (A, B, D) Nuclei are stained with DAPI (blue). (E) A graph showing results of quantification of microvessel pericyte coverage index (MPI) in atrial samples of all patients as described in Methods. RA–right appendage (n = 8), LA–left appendage (n = 9), LAt–left atrium (n = 7), LA + LAt (n = 16).

To evaluate possible differences in activity of angiogenic process in atrial myocardium of patients with AF and in SR, microvessel pericyte coverage of capillaries in atrial tissue samples was analyzed. Expression of two most commonly used markers: desmin and smooth muscle actin was assessed. While desmin was immunopositive in cardiomyocytes, capillaries were devoid of desmin signal (Fig 6A and 6B). On the other hand, we could detect smooth muscle actin-positive pericytes surrounding capillaries as well as immunoreactive smooth muscle cells in the wall of arterioles, small arteries and veins within atrial myocardium (Fig 6B–6D). Quantitative analysis of microvessel pericyte coverage index showed that there was no significant difference between AF and SR patient groups (S3 Table). A comparison of microvessel pericyte coverage index in samples from different anatomical locations showed that there is similar pericyte coverage in left and right atrial myocardium (Fig 6E).

## Discussion

### Main findings

This study that aimed at a comprehensive analysis of atrial interstitium in patients undergoing cardiac surgery for significant cardiovascular disease with and without previous history of AF provided results that can be summarized as follows: 1) despite the fact that subjects with AF had significantly larger atria and were older, no significant differences in the amount of collagen I and III, and of elastin were observed in their atrial interstitium in comparison with patients in SR group, 2) both patient groups also did not differ in expression of atrial myocardial VEGF or in microvascular density, 3) tissue content of elastin, collagen I and collagen III and VEGF expression in the right atrium were significantly higher than in samples obtained from the left atrium, and 4) microvascular density was greater in the left atrial myocardium compared to the right atrial myocardium.

### Structural remodeling

Despite a plethora of studies published in the past that focused on specific morphological features of human atria during AF, this paper presents a study based on bioptic samples from both left and right atria and compares two cardiac patient groups with and without long-term persistent AF. We could confirm that both atria are dilated in patients with AF, while there is no significant difference in the left ventricular parameters as reported also in other studies with similar patient groups [11, 12]. There is an increased risk of AF in patients with heart failure [13]. However, patients included in our AF and SR groups did not have significant differences in their left ventricular function or their NYHA class. This study suggests that interstitial fibrosis and other morphological changes in atrial tissue are rather linked to structural heart disease than to AF per se. This is a contradiction to a common belief about AF-related structural remodeling of the atrial tissue. The explanation could be that other factors play role in the etiopathogenesis of AF such as the presence of triggers and/or modulation by the autonomic nervous system, inflammation, etc.

In addition, earlier studies that showed more significant morphological changes in atrial tissue of AF patients used different methodology for collagen detection and quantification [6, 14, 15]. In the present comprehensive study, specific fibrous components of extracellular matrix—collagen I, collagen III and elastin—were quantified separately. Surprisingly, their volume fraction was found similar in patients undergoing cardiac surgery whether they did have or did not have AF. Our results are supported by other recently published thorough morphological analysis of human atrial myocardium obtained during autopsy that included histomorphometry of routinely stained samples [12]. Comparing subjects with and without AF, the study showed similar level of fibrosis when AF and SR groups were compared. Similarly, in another paper comparing both left and right atrial appendages in biopsies from patients with mitral valve disease, a significantly higher level of fibrosis in AF patients was found only in the right appendage, but no difference between AF and SR groups was found when samples from the left appendages were compared [16]. Our study extended such observation also to the content of elastin in patients with and without AF. Importantly, aiming at possible regional differences, we found several fold higher elastin volume fraction in the right atrium compared to the left atrium when samples from all patients were pooled together. Similar, but less pronounced differences were also found in the case of collagen I and collagen III volume fractions. Such observed higher volume fraction of extracellular matrix proteins in the right atrium is an original observation that requires further studies. It might be a consequence of different biomechanical stimuli for fibroblasts or a result of different origins of endomysial cells due to complicated heart development [17]. However, a role of elastin in the myocardium is not fully explained [9].

### Atrial microvasculature and VEGF expression

As shown by our data, the expression of VEGF can be regularly detected in atrial samples of patients regardless of their heart rhythm. Importantly, there was no significant difference in myocardial VEGF expression between patients with AF and SR. In this respect, the mere presence of VEGF suggests active angiogenesis or at least the effect on maintenance of vascular bed in the myocardium of patients with various heart pathologies. One study on biopsies from right atrial appendages of patients undergoing routine open heart surgery reported on an increased expression of VEGF in AF patients [10]. However, these authors did not specify the localization of VEGF expression. Another study, performed also on biopsies of right atrial appendages, demonstrated an elevated VEGF expression on mRNA and protein level in the whole thickness of the atrial wall [11]. When these authors localized VEGF expression using immunofluorescence, they reported an elevation of VEGF signal in endothelium of arteries within the atrial wall during AF. This is in contrast with our observation of VEGF expression also in other structures such as capillaries, cardiomyocytes and epicardium.

In line with similar VEGF expression in atria from patients with SR and AF in this study, we also found comparable microvascular density in atrial myocardium of both patient groups. Similar finding was reported in one above mentioned study. The authors discovered no difference in microvascular density in the right atrial appendage biopsies of patients undergoing open heart surgery both with and without AF [10]. Capillary density was also quantified in the study performed on autopsy samples of atria from patients with AF and SR and again, no difference was detected between these patient groups [18].

Another novel observation of our study is anatomical variability both in VEGF expression by cardiomyocytes and microvascular density. Higher VEGF expression of right atrial cardiomyocytes could reflect a need for stronger angiogenic stimulation as cardiomyocytes are according to our data separated from each other by a higher amount of extracellular matrix fibers in endomysium that has less dense microvascular network. Another possibility how to further characterize myocardial vasculature is to determine pericyte coverage index of microvessels. Our findings demonstrate again similar level of microvessel pericyte coverage index in myocardia of patients regardless of heart rhythm. Microvessel pericyte coverage index found in our study is comparable with findings in normal rat hearts that have microvessel pericyte coverage index around 84% [19]. Although there are no physiological human data available, we can conclude that microvasculature of atrial myocardium of patients in the present study is well-equipped with mural cells.

### Study limitations

The study shares several limitations common to a histopathological approach that uses material obtained from living patients during surgery. Our patient groups were relatively small and this fact might have influenced our quantitative results. For obvious ethical reasons, bioptic samples can be only of a limited size and number, and can be safely harvested only from certain locations within the atrial wall. As our knowledge of myocardial architecture expands, it is becoming increasingly obvious that there is a high level of variability even within a single atrium [20]. We analyzed above mentioned parameters on single 2D tissue sections and it would be more advantageous to analyze the whole 3D volume reconstruction e.g. from z-stacks of histological sections so that information on fibrosis distribution was more complete. A more systematic sampling is possible in autoptic studies but this material usually comes from patients with even more complicated history of cardiovascular disease and other co-morbidities. One can see another limitation of the study that we compared slightly different groups of cardiac patients with a higher representation of valvular heart disease in the AF group. In such patients, the fibrosis might be expected as a result of underlying disease. However, even patients with high extent of atrial fibrosis are apparently able to maintain SR. Despite the fact that AF patients were older, had more mitral regurgitation and higher atrial volumes, no difference was found in quantitative analysis of determinants of structural remodeling of atrial tissue. It would have been more instructive to analyze bioptic samples of atrial tissue from patients with lone AF in comparison with control group of subjects without AF; however, such study cannot be for apparent ethical reasons performed.

### Conclusions

Comprehensive analysis of bioptic samples of atrial tissue in a cohort of cardiac patients undergoing open heart surgery revealed that the interstitium has variable morphology, especially in terms of the amount and composition of extracellular matrix. However, we could not confirm a higher level of fibrosis specific for AF. This finding suggests that interstitial fibrosis and other morphological changes in atrial tissue are rather linked to structural heart disease than to AF per se. Whether such patient will develop AF, depends most probably on additional presence of triggers and modulating factors. In addition, we showed that differences in the degree of structural remodeling between left and right atrium may be more important for etiopathogenesis of AF. Regional variation in matrix fiber content, microvascular density and VEGF expression deserve further investigation.

## Supporting Information

S1 Table Characteristics of patients with AF and patients in SR. (DOC)

S2 Table Characterization of antibodies used in the study. (DOC)

S3 Table Histomorphometry of samples from patients with atrial fibrillation and sinus rhythm. Table shows the results of quantitative analysis of several morphological parameters as a comparison between samples from patients with atrial fibrillation (AF) or sinus rhythm (SR). Details of histomorphometry are described in Methods. The values are expressed as the mean±SD. Comparison between both groups was performed using a non-parametric test—Mann–Whitney U test. A value of P < 0.05 was considered significant.
(DOC)

S4 Table VEGF expression in samples from patients with atrial fibrillation and sinus rhythm. Table shows the results of semiquantitative analysis of VEGF expression. Details of histomorphometry are described in Methods. Table shows the intensity of positive cardiomyocytes, fat cells, mesothelial cells and capillaries. The intensity score consisted of four grades (0–3), with 0 representing no staining and 3 denoting the maximum staining effect. The values are expressed as the mean±SD. Comparison between both groups was performed using a non-parametric test—Mann–Whitney U test. A value of P < 0.05 was considered significant. ND = not determined.
(DOC)

## References

[1] Heeringa J, van der Kuip DA, Hofman A, Kors JA, van Herpen G, Stricker BH, et al Prevalence, incidence and lifetime risk of atrial fibrillation: the Rotterdam study. Eur Heart J. 2006 4;27(8):949–53. 16527828 10.1093/eurheartj/ehi825

[2] Wyse DG, Van Gelder IC, Ellinor PT, Go AS, Kalman JM, Narayan SM, et al Lone atrial fibrillation: does it exist? J Am Coll Cardiol. 2014 5 6;63(17):1715–23. 10.1016/j.jacc.2014.01.023 24530673 PMC4008692

[3] Wakili R, Voigt N, Kaab S, Dobrev D, Nattel S. Recent advances in the molecular pathophysiology of atrial fibrillation. J Clin Invest. 2011 8;121(8):2955–68. 10.1172/JCI46315 21804195 PMC3148739

[4] Allessie M, Ausma J, Schotten U. Electrical, contractile and structural remodeling during atrial fibrillation. Cardiovasc Res. 2002 5;54(2):230–46. 12062329 10.1016/s0008-6363(02)00258-4

[5] Frustaci A, Chimenti C, Bellocci F, Morgante E, Russo MA, Maseri A. Histological substrate of atrial biopsies in patients with lone atrial fibrillation. Circulation. 1997 8 19;96(4):1180–4. 9286947 10.1161/01.cir.96.4.1180

[6] Boldt A, Wetzel U, Lauschke J, Weigl J, Gummert J, Hindricks G, et al Fibrosis in left atrial tissue of patients with atrial fibrillation with and without underlying mitral valve disease. Heart. 2004 4;90(4):400–5. 15020515 10.1136/hrt.2003.015347 PMC1768173

[7] Ferreira JP, Santos M. Heart failure and atrial fibrillation: from basic science to clinical practice. Int J Mol Sci. 2015;16(2):3133–47. 10.3390/ijms16023133 25647414 PMC4346884

[8] De Jong AM, Maass AH, Oberdorf-Maass SU, Van Veldhuisen DJ, Van Gilst WH, Van Gelder IC. Mechanisms of atrial structural changes caused by stretch occurring before and during early atrial fibrillation. Cardiovasc Res. 2011 3 1;89(4):754–65. 10.1093/cvr/cvq357 21075756

[9] Fomovsky GM, Thomopoulos S, Holmes JW. Contribution of extracellular matrix to the mechanical properties of the heart. J Mol Cell Cardiol. 2010 3;48(3):490–6. 10.1016/j.yjmcc.2009.08.003 19686759 PMC2823835

[10] Gramley F, Lorenzen J, Jedamzik B, Gatter K, Koellensperger E, Munzel T, et al Atrial fibrillation is associated with cardiac hypoxia. Cardiovasc Pathol. 2010 Mar-Apr;19(2):102–11. 10.1016/j.carpath.2008.11.001 19211267

[11] Ogi H, Nakano Y, Niida S, Dote K, Hirai Y, Suenari K, et al Is structural remodeling of fibrillated atria the consequence of tissue hypoxia? Circ J. 2010 9;74(9):1815–21. 20631454 10.1253/circj.cj-09-0969

[12] de Oliveira IM, Oliveira BD, Scanavacca MI, Gutierrez PS. Fibrosis, myocardial crossings, disconnections, abrupt turns, and epicardial reflections: do they play an actual role in human permanent atrial fibrillation? A controlled necropsy study. Cardiovasc Pathol. 2013 Jan-Feb;22(1):65–9. 10.1016/j.carpath.2012.06.001 22917538

[13] Darby AE, Dimarco JP. Management of atrial fibrillation in patients with structural heart disease. Circulation. 2012 2 21;125(7):945–57. 10.1161/CIRCULATIONAHA.111.019935 22354975

[14] Kostin S, Klein G, Szalay Z, Hein S, Bauer EP, Schaper J. Structural correlate of atrial fibrillation in human patients. Cardiovasc Res. 2002 5;54(2):361–79. 12062341 10.1016/s0008-6363(02)00273-0

[15] Polyakova V, Miyagawa S, Szalay Z, Risteli J, Kostin S. Atrial extracellular matrix remodelling in patients with atrial fibrillation. J Cell Mol Med. 2008 Jan-Feb;12(1):189–208. 10.1111/j.1582-4934.2008.00219.x 18194448 PMC3823481

[16] Anne W, Willems R, Roskams T, Sergeant P, Herijgers P, Holemans P, et al Matrix metalloproteinases and atrial remodeling in patients with mitral valve disease and atrial fibrillation. Cardiovasc Res. 2005 9 1;67(4):655–66. 15913581 10.1016/j.cardiores.2005.04.016

[17] Porter KE, Turner NA. Cardiac fibroblasts: at the heart of myocardial remodeling. Pharmacol Ther. 2009 8;123(2):255–78. 10.1016/j.pharmthera.2009.05.002 19460403

[18] Platonov PG, Mitrofanova LB, Orshanskaya V, Ho SY. Structural abnormalities in atrial walls are associated with presence and persistency of atrial fibrillation but not with age. J Am Coll Cardiol. 2011 11 15;58(21):2225–32. 10.1016/j.jacc.2011.05.061 22078429

[19] Tilton RG, Kilo C, Williamson JR. Pericyte-endothelial relationships in cardiac and skeletal muscle capillaries. Microvasc Res. 1979 11;18(3):325–35. 537510 10.1016/0026-2862(79)90041-4

[20] Zhao J, Butters TD, Zhang H, Pullan AJ, LeGrice IJ, Sands GB, et al An image-based model of atrial muscular architecture: effects of structural anisotropy on electrical activation. Circ Arrhythm Electrophysiol. 2012 4;5(2):361–70. 10.1161/CIRCEP.111.967950 22423141

