# Supplementary material for: Bioptic Study of Left and Right Atrial Interstitium in Cardiac Patients with and without Atrial Fibrillation: Interatrial but Not Rhythm-Based Differences
Source: PLoS One. 2015 Jun 12;10(6):e0129124. doi: 10.1371/journal.pone.0129124 (PMC4466374; doi:10.1371/journal.pone.0129124)
Supplement: S1 Table — (DOC) [file pone.0129124.s001.doc]

**Supporting Information Table 1. Characteristics of patients with AF and patients in SR**

**Total**

**AF**

**SR**

**P value**

(n=46)

(n=19)

(n=27)

Age (years)

.01

Sex (m/f)

25/21

13/6

12/15

Height (cm)

Weight (kg)

n.s.

Obesity

19(41.3%)

7(36.8%)

Diabetes mellitus

10(21.7%)

4(21.1%)

6(22.2%)

Coronary artery disease

32(69.6%)

8(42.1%)

24(88.9%)

Mitral regurgitation grade (0-4)

.0001

LV diameter (mm)

LV EF (%)

LA volume (ml)

CHA2DS2-VASc score

3.35±1.86

1.79±1.08

3.30±1.30

1.57±1.37

2.31±0.54

1.41±0.93

2.33±0.64

3.42±1.50

**Characteristic**

93.4±51.4

121.3±62.5

73.0±28.5

.001

51.6±10.4

49.5±11.7

53.1±9.3

n.s.

54.0±7.0

55.4±8.0

53.0±6.1

n.s.

Aortic valve stenosis grade (0-4)

1.21±1.61

1.06±1.42

1.32±1.74

n.s.

Tricuspid valve regurgitation grade (0-4)

1.74±0.82

2.40±0.87

1.34±0.45

<.0001

2.18±1.03

2.91±1.02

1.72±0.74

Arterial hypertension

37(80.4%)

16(84.2%)

21(77.8%)

84±19

84±21

83±15

169±10

170±10

169±10

65±9

69±7

62±9

RA volume (ml)

71.0±33.0

máš Kučera Vám,

ěstí.

oskop84.2±39.5

61.3±23.8

12(44.4%)

n.s.

n.s.

n.s.

n.s.

n.s.

n.s.

n.s.

.001

.05

CHADS2 score

Atrial fibrillation duration (years)

8.3±11.1

---

---

---

2.32±0.59

n.s.

NYHA class (I-IV)

**Type of surgery**

8(42.1%)

CABG

32(69.6%)

24(88.9%)

.001

Mitral valve repair or replacement

12(26.1%)

4(14.8%)

8(42.1%)

.05

Aortic valve replacement

17(37.0%)

8(42.1%)

9(42.9%)

n.s.

LV, left ventricle; EF, ejection fraction; LA, left atrium; RA, right atrium; CABG, coronary artery bypass surgery; CHADS2 (The Congestive heart failure, Hypertension, Age ˃75, Diabetes mellitus, Stroke), CHA2DS2-VASc (The Congestive heart failure, Hypertension, Age ˃75, Diabetes mellitus, Stroke, Vascular disease, Age 65-74 years, Sex category) stroke risk assessment in Atrial Fibrillation; n.s.: not significant. Values are expressed as average ±SD.
