# Supplementary material for: Bioptic Study of Left and Right Atrial Interstitium in Cardiac Patients with and without Atrial Fibrillation: Interatrial but Not Rhythm-Based Differences
Source: PLoS One. 2015 Jun 12;10(6):e0129124. doi: 10.1371/journal.pone.0129124 (PMC4466374; doi:10.1371/journal.pone.0129124)
Supplement: S2 Table — (DOC) [file pone.0129124.s002.doc]

**Supporting Information Table 2.** **Characterization of antibodies used in the study**

**Antibody**

**Abbreviation**

**Catalog ID/Lot**

**ID**

**Dilution**

**Retrieval**

**Clone/Isotype**

**Produced by**

Monoclonal Mouse Anti-Human

Smooth Muscle Actin

SMA

M0851

1:100

Tris buffer +EDTA

1A4 IgG2a

DakoCytomation,

Glostrup, Denmark

Monoclonal Mouse Anti-Human

Vascular Endothelial Growth

Factor

VEGF

M7273

1:100

Citrate buffer

VG1 IgG1

DakoCytomation,

Glostrup, Denmark

Monoclonal Mouse Anti-Human

Elastin

ELAST

E4013/019K4828

1:250

TRYPSIN

BA-4 IgG1

Sigma-Aldrich, USA

Monoclonal Mouse Anti-Human

Desmin

DESM

IR606

1:100

Tris buffer +EDTA

D33 IgG1

DakoCytomation,

Glostrup, Denmark

Collagen I

(Rabbit Anti-Human polyclonal)

Collagen III

(Rabbit Anti-Human polyclonal)

Abcam,

CambridgeScience,

Cambridge UK

Col III

LS-B693/36517

1:1000

Citrate buffer

LifeSpan BioSciences,

USA

Col I

ab34710/GR3358 5-3

1:1000

Citrate buffer
