# Supplementary material for: Bioptic Study of Left and Right Atrial Interstitium in Cardiac Patients with and without Atrial Fibrillation: Interatrial but Not Rhythm-Based Differences
Source: PLoS One. 2015 Jun 12;10(6):e0129124. doi: 10.1371/journal.pone.0129124 (PMC4466374; doi:10.1371/journal.pone.0129124)
Supplement: S3 Table — Table shows the results of quantitative analysis of several morphological parameters as a comparison between samples from patients with atrial fibrillation (AF) or sinus rhythm (SR). Details of histomorphometry are described in Methods. The values are expressed as the mean±SD. Comparison between both groups was performed using a non-parametric test—Mann–Whitney U test. A value of P < 0.05 was considered significant. (DOC) [file pone.0129124.s003.doc]

**Supporting Information Table 3. Histomorphometry of samples from patients with atrial fibrillation and sinus rhythm**

Table shows the results of quantitative analysis of several morphological parameters as a comparison between samples from patients with atrial fibrillation (AF) or sinus rhythm (SR). Details of histomorphometry are described in Methods. The values are expressed as the mean±SD. Comparison between both groups was performed using a non-parametric test - Mann–Whitney U test. A value of *P* < 0.05 was considered significant.

CI-VF = collagen I volume fraction; CIII-VF = collagen III volume fraction; EVF = elastin volume fraction; MVD = microvessel density; MPI = microvessel pericyte coverage index; SR = sinus rhythm; AF = atrial fibrillation
