# Supplementary material for: Bioptic Study of Left and Right Atrial Interstitium in Cardiac Patients with and without Atrial Fibrillation: Interatrial but Not Rhythm-Based Differences
Source: PLoS One. 2015 Jun 12;10(6):e0129124. doi: 10.1371/journal.pone.0129124 (PMC4466374; doi:10.1371/journal.pone.0129124)
Supplement: S4 Table — Table shows the results of semiquantitative analysis of VEGF expression. Details of histomorphometry are described in Methods. Table shows the intensity of positive cardiomyocytes, fat cells, mesothelial cells and capillaries. The intensity score consisted of four grades (0–3), with 0 representing no staining and 3 denoting the maximum staining effect. The values are expressed as the mean±SD. Comparison between both groups was performed using a non-parametric test—Mann–Whitney U test. A value of P < 0.05 was considered significant. ND = not determined. (DOC) [file pone.0129124.s004.doc]

**Supporting Information Table 4. VEGF expression in samples from patients with atrial fibrillation and sinus rhythm**

Table shows the results of semiquantitative analysis of VEGF expression. Details of histomorphometry are described in Methods.

Table shows the intensity of positive cardiomyocytes, fat cells, mesothelial cells and capillaries. The intensity score consisted of four grades

(0–3), with 0 representing no staining and 3 denoting the maximum staining effect. The values are expressed as the mean±SD. Comparison between both groups was performed using a non-parametric test - Mann–Whitney U test. A value of *P* < 0.05 was considered significant. ND = not determined.

VEGF = vascular endothelial growth factor; SR = sinus rhythm; AF = atrial fibrillation
